# Crystal structure and Hirshfeld-surface analysis of 1-(4-fluoro­phen­yl)-3,3-bis­(methyl­sulfan­yl)prop-2-en-1-one

**DOI:** 10.1107/S2056989025004189

**Published:** 2025-05-13

**Authors:** Thaluru M. Mohan Kumar, Besagarahally L. Bhaskar, Papegowda Bhavya, Thayamma R. Divakara, Holehundi J. Shankara Prasad, Hemmige S. Yathirajan, Sean Parkin

**Affiliations:** a Department of Physical Sciences, Amrita School of Engineering, Amrita Vishwa Vidyapeetham, Bengaluru-560 035, India; b Department of Applied Sciences, New Horizon College of Engineering, Bengaluru-560 103, India; c Department of Chemistry, T. John Institute of Technology, Bengaluru-560 083, India; d Department of Chemistry Yuvaraja's College University of Mysore,Mysore-570 005 India; e Department of Studies in Chemistry University of Mysore, Manasagangotri Mysuru-570 006 India; f Department of Chemistry University of Kentucky,Lexington KY 40506-0055 USA; Institute of Chemistry, Chinese Academy of Sciences

**Keywords:** crystal structure, Hirshfeld-surface analysis, chalcone derivative

## Abstract

The title compound, C_11_H_11_FOS_2_, is a fluorinated chalcone derivative with potential applications in medicinal chemistry and functional materials. The mol­ecular structure includes a planar 4-fluoro­phenyl ring linked by a carbonyl group and an ethenyl spacer to an approximately planar bis­(methyl­sulfan­yl) moiety (r.m.s. deviations from planarity are 0.0106 and 0.0315 Å, respectively). These planar groups are twisted relative to each other, subtending a dihedral angle of 32.23 (4)°. The crystal packing lacks classical hydrogen bonds or aromatic π-stacking, but mol­ecules are connected through weaker C—H⋯O and C—H⋯S contacts into layers parallel to the *ab* plane and tapes extending along the *b*-axis direction. The 4-fluoro­phenyl groups on adjacent tapes inter­digitate. Hirshfeld surface analysis shows that the majority (>90%) of inter­molecular contacts involve hydrogen atoms.

## Abstract 2

The crystal structure and a Hirshfeld-surface analysis of the chalcone derivative 1-(4-fluoro­phen­yl)-3,3-bis­(methyl­sulfan­yl)prop-2-en-1-one are presented.

## 1. Chemical context

1-(4-Fluoro­phen­yl)-3,3-bis­(methyl­sulfan­yl)prop-2-en-1-one (**I**) is a fluorinated chalcone derivative with potential applications in medicinal and functional materials chemistry. Natural and synthetic chalcones have been widely utilized for their broad spectrum of biological activities, which include anti-microbial, anti-cancer, anti-diabetic, anti-inflammatory, anti-oxidant, anti-parasitic, and neuroprotective effects (Lin *et al.*, 2002 ▸; Bhat *et al.*, 2005 ▸; Trivedi *et al.*, 2007 ▸; Lahtchev *et al.*, 2008 ▸; Aneja *et al.*, 2018 ▸). The presence of fluorine may enhance inter­actions with biological systems, potentially inhibiting enzyme activity or receptors involved in disease processes. In this context, fluorinated chalcone derivatives have shown notable bioactivity (Nakamura *et al.*, 2002 ▸). The significance of the chalcone scaffold in medicinal chemistry is well established, having been identified as a ‘privileged structure’ with considerable therapeutic potential (Zhuang *et al.*, 2017 ▸). Several reviews have explored the synthesis, structural diversity, and biological relevance of chalcones and their derivatives, including their roles as anti-infective agents and enzyme inhibitors (Nowakowska, 2007 ▸; Elkanzi *et al.*, 2022 ▸; de Mello *et al.*, 2018 ▸; Opletalova & Sedivy, 1999 ▸). The methyl­sulfanyl groups also contribute to its chemical reactivity (Nakamura *et al.*, 2002 ▸), suggesting this class of compounds as promising candidates in the development of advanced functional materials. Examples include applications in optical data storage systems (Corredor *et al.*, 2007 ▸), electronics and coatings (Belahlou *et al.*, 2020 ▸) as well as non-linear optical (NLO) materials (Xu *et al.*, 2020 ▸).
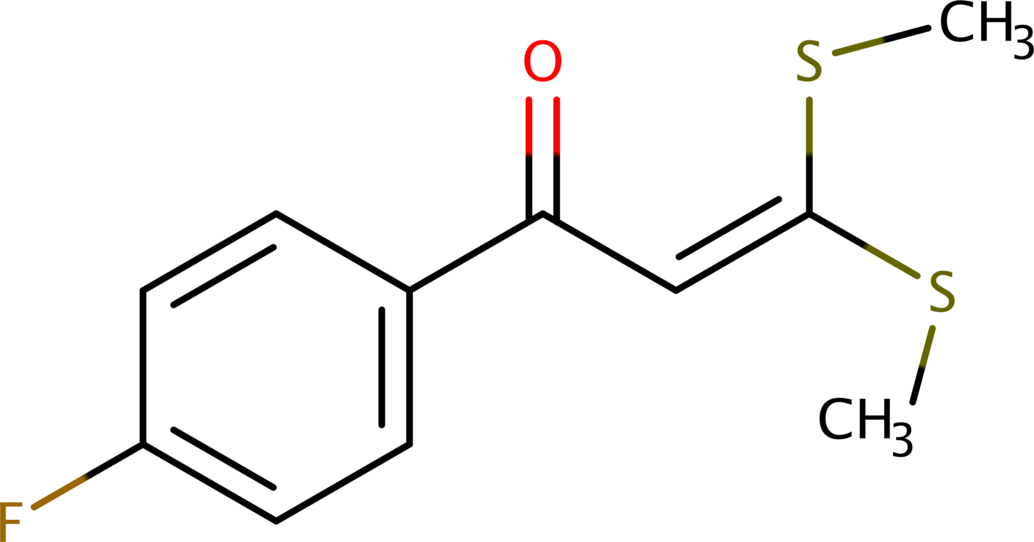


In light of the importance of chalcones and their derivatives in several areas of chemistry, physics, medicine, pharmaceuticals and biology, this paper reports the crystal structure and a Hirshfeld-surface analysis of **I**.

## 2. Structural commentary

**Figure 1:**
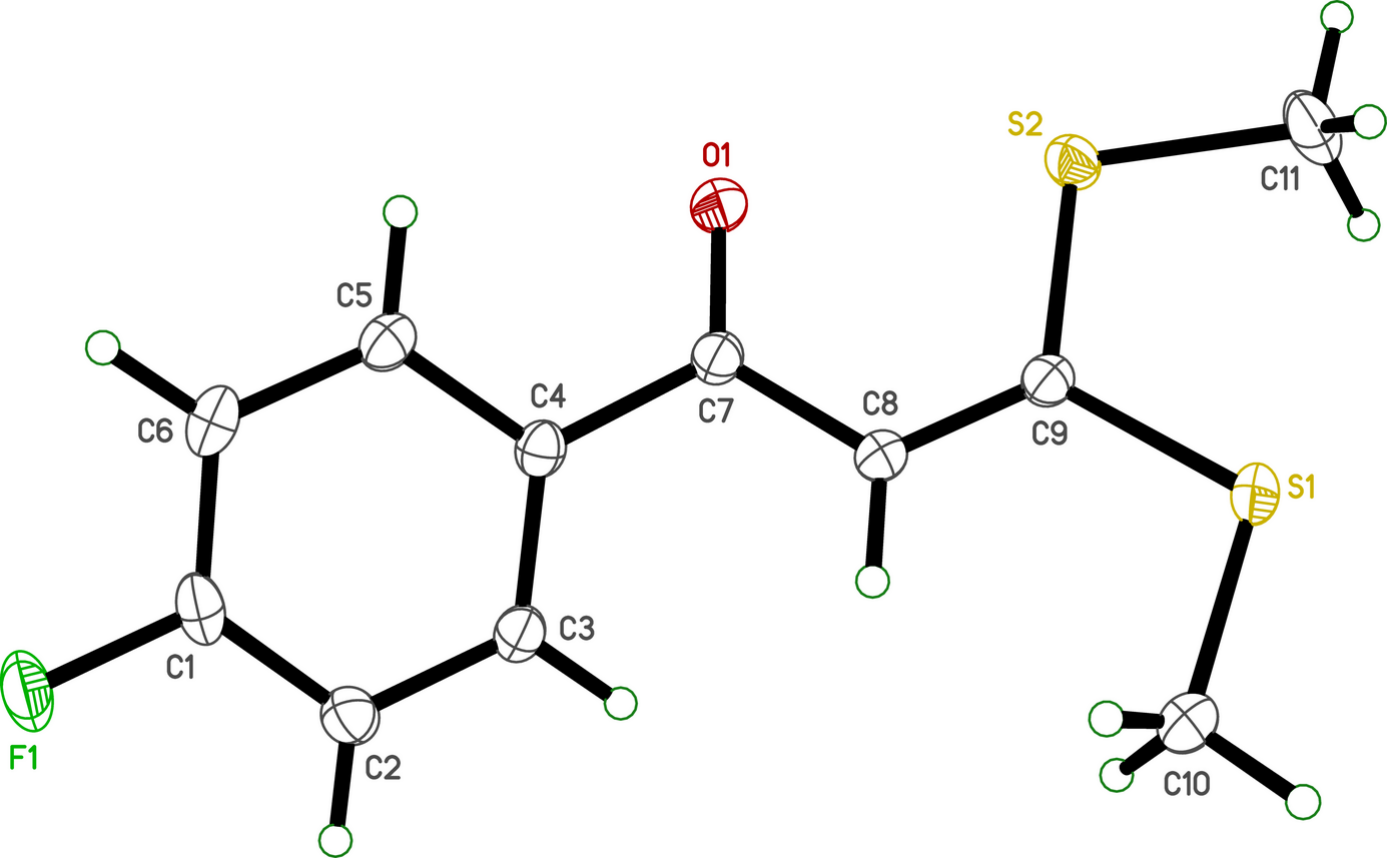
An ellipsoid plot (50% probability) of **I**. Hydrogen atoms are drawn as small arbitrary circles.

The mol­ecular structure of **I** consists of a 4-fluoro­phenyl ring bonded to a carbonyl group, which in turn is attached *via* an ethenyl linker to a bis­(methyl­sulfan­yl) moiety, as shown in Fig. 1 ▸. All bond lengths and angles in **I** fall within normal ranges. The overall geometry is essentially that of two planar groups twisted with respect to each other about the C4—C7 bond [torsion C5—C4—C7—O1 = −28.68 (16)°], with a smaller twist about the C7—C8 bond [O1—C7—C8—C9 = 4.91 (18)°]. These two planar moieties are the 4-fluoro­phenyl group, which is necessarily flat (r.m.s deviation = 0.0106 Å), and the bis­(methyl­sulfan­yl)propenone group (atoms C7, O1, C8, C9, S1, S2, C10, C11), which is also close to planarity [r.m.s. = 0.0315 Å, maximum deviation = 0.0412 (1) Å at C9]. The dihedral angle between these two planar regions is 32.23 (4)°. Representative torsion angles are given in Table 1 ▸.

## 3. Supra­molecular features

**Figure 2:**
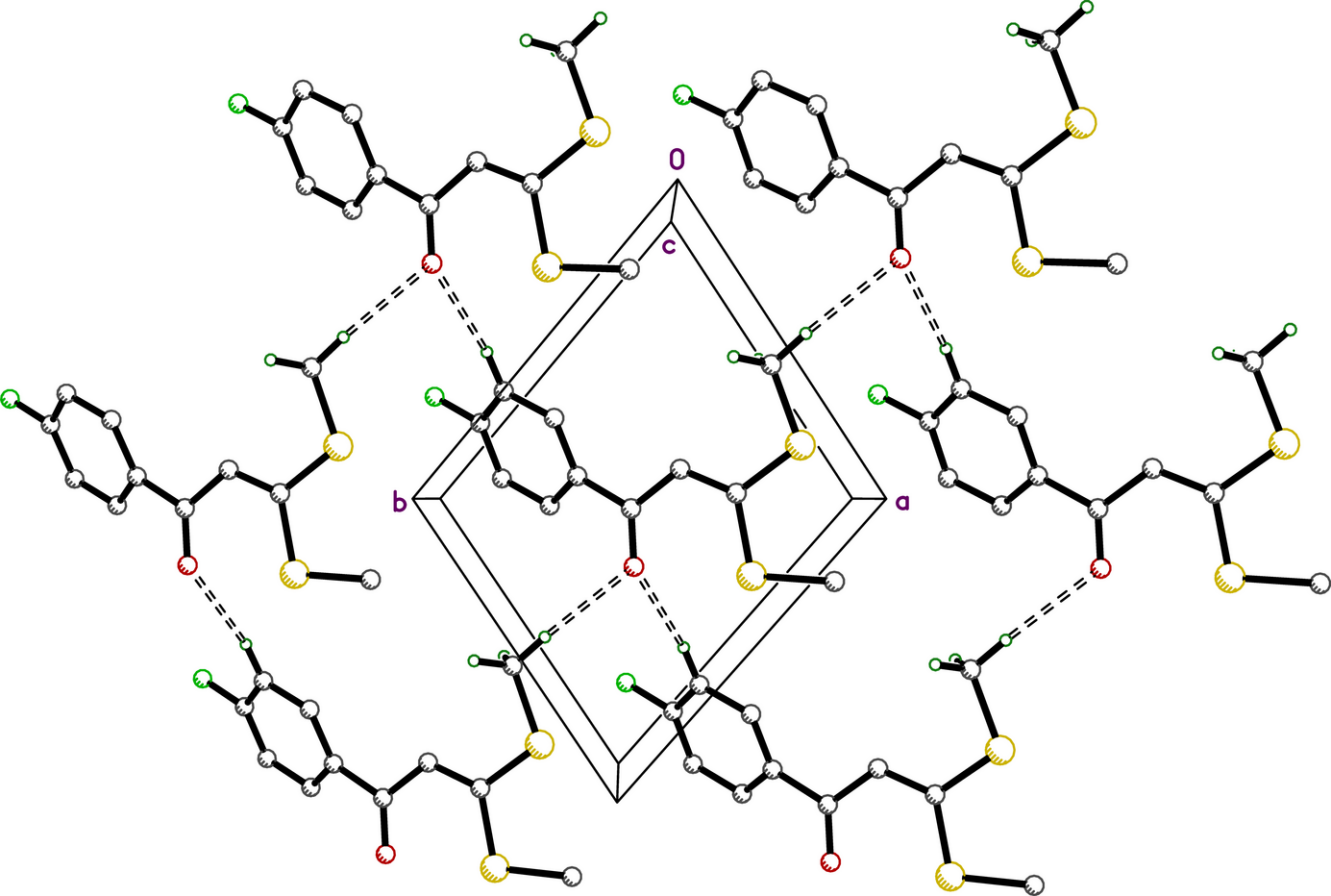
A partial packing plot of **I** viewed normal to the *ab* plane. C—H⋯O inter­actions that connect the mol­ecules into layers are shown as open dashed lines.

**Figure 3:**
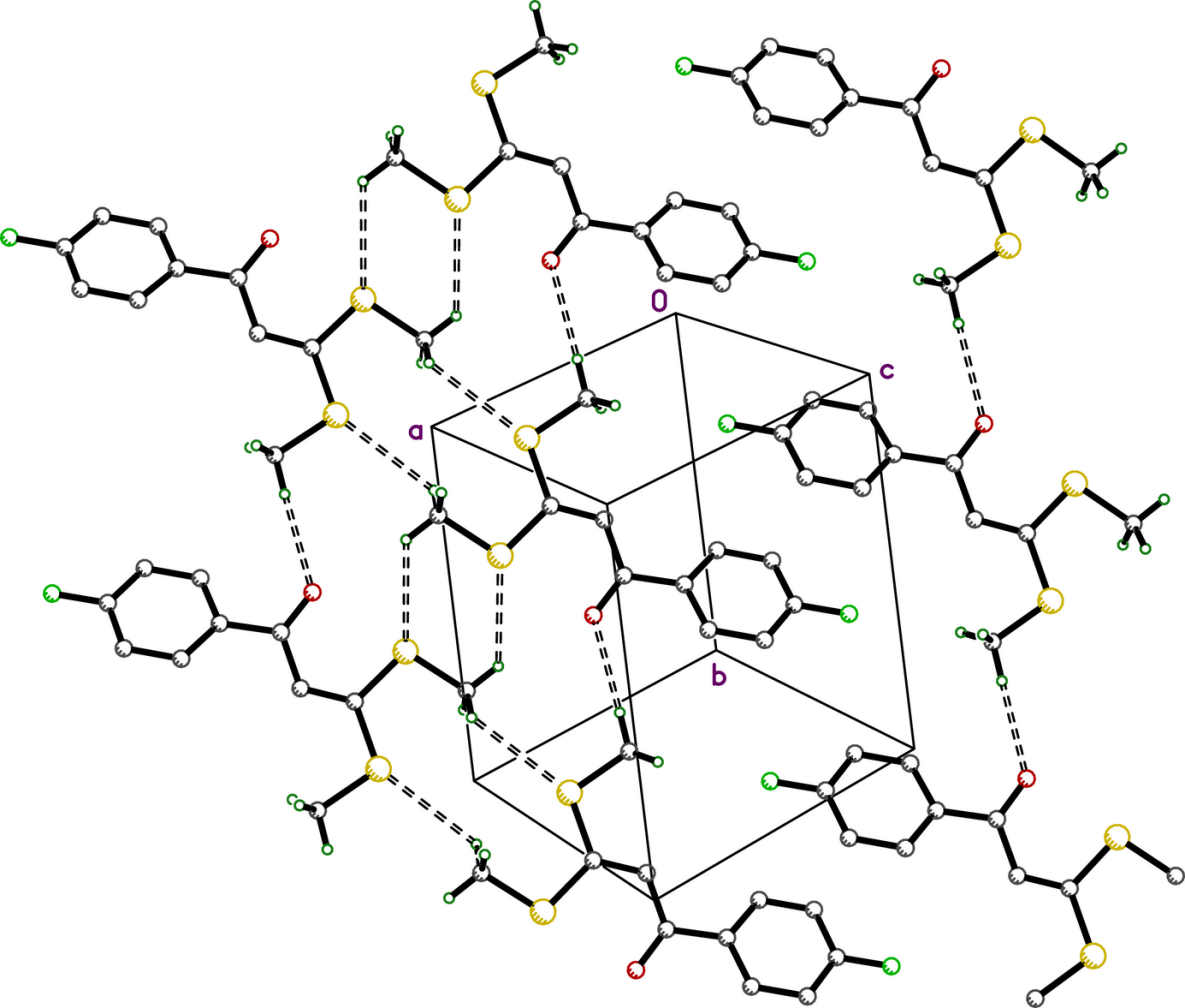
A partial packing plot of **I** viewed approximately perpendicular to [307], showing tapes of mol­ecules inter­acting *via* C—H⋯S and C—H⋯O contacts. The 4-fluoro­phenyl groups of adjacent tapes inter­digitate.

**Figure 4:**
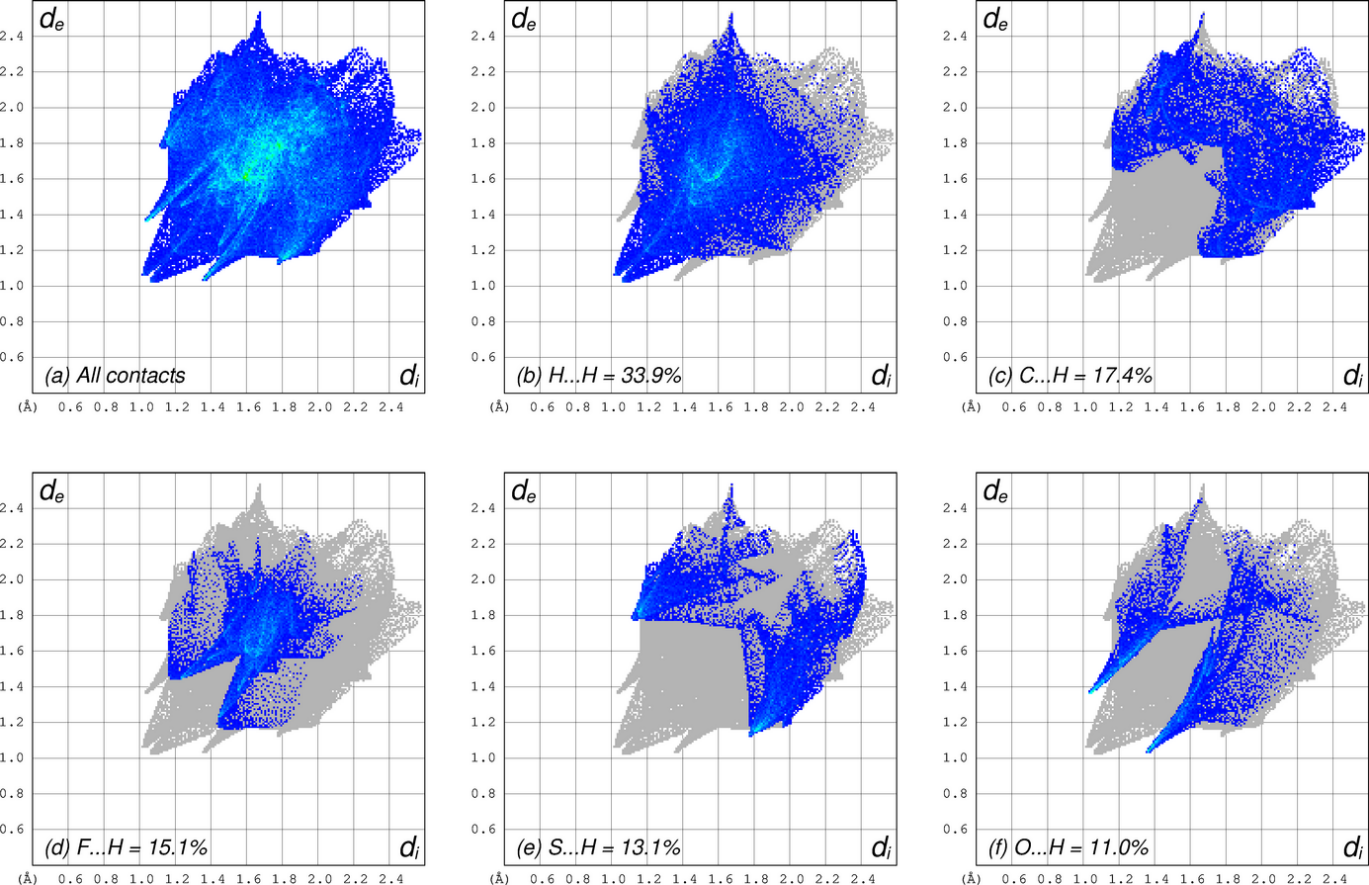
Hirshfeld-surface two-dimensional fingerprint plots showing (*a*) all contacts, (*b*) H⋯H contacts, (*c*) C⋯H contacts, (*d*) F⋯H contacts, (*e*) S⋯H contacts, and (*f*) O⋯H contacts.

There are no conventional hydrogen bonds in the crystal structure of **I**, nor any π–π stacking of aromatic rings. There are, however, several weaker C—H⋯O and C—H⋯S close contacts, which are summarized in Table 2 ▸. Contacts C2—H2⋯O1^i^ and C10—H10*C*⋯O1^ii^ [*d_D⋯A_* = 3.4530 (15) and 3.4724 (16) Å] join the mol­ecules into layers parallel to the *ab* plane, as shown in Fig. 2 ▸. The C11—H11*A*⋯S1^iii^ and C11—H11*C*⋯S2^iv^ contacts [*d_D⋯A_* = 3.6032 (14) and 3.6292 (13) Å; all symmetry codes as per Table 2 ▸] join mol­ecules into tapes that extend along the *b*-axis direction, roughly parallel to [307]. The 4-fluoro­phenyl groups of adjacent tapes inter­digitate. These inter­actions are shown in Fig. 3 ▸. A Hirshfeld surface analysis conducted using *Crystal Explorer 21* (Spackman *et al.*, 2021 ▸) shows that over 90% of inter­molecular contacts involve hydrogen. The 2D fingerprint plots of the five most abundant atom–atom contacts are shown in Fig. 4 ▸.

## 4. Database survey

A search of the Cambridge Structural Database (CSD, v5.46, November 2024; Groom *et al.*, 2016 ▸) using a fragment consisting of **I** but with the fluorine position set to ‘any atom’, returned 29 hits, three of which were duplicates. However, only six of these had a hydrogen attached to the central carbon of the propene moiety (*i.e*., C8 in **I**). Of these six, KUQQEV (Madan Kumar *et al.*, 2020 ▸) had chlorine at the *ortho* position of the benzene ring, while WENVIV (Liao *et al.*, 2006 ▸) had a 2,6-dimethyl-3,5-di­nitro-4-^*t*^Bu phenyl group. The remaining four structures with para-substituted phenyl groups are thus the most similar to **I**. Entry MTBZOE (Mellor & Nyburg, 1971 ▸) has *X* = H, OCUSEN (Verma & Singh, 2016 ▸) has *X* = OMe, LUYXAH (Hussain *et al.*, 2018 ▸) has *X* = CF_3_, and AFOMEP (Yu *et al.*, 2013 ▸) has *X* = NO_2_.

## 5. Synthesis and crystallization

Synthesis of **I** was as described in the literature procedure by Huynh *et al.* (2025 ▸). The product obtained was purified by column chromatography and recrystallized from chloro­form by slow evaporation, yielding reddish brown crystals (m.p.: 358–359 K).

## 6. Refinement

Crystal data, data collection and structure refinement details are summarized in Table 3 ▸. All hydrogen atoms were found in difference-Fourier maps, but subsequently included in the refinement using riding models, with constrained distances set to 0.95 Å (C*sp*^2^—H) and 0.98 Å (*R*CH_3_). *U*_iso_(H) parameters were set to values of either 1.2*U*_eq_ or 1.5*U*_eq_ (*R*CH_3_) of the attached atom.

## Supplementary Material

Crystal structure: contains datablock(s) I, global. DOI: 10.1107/S2056989025004189/nx2025sup1.cif

Structure factors: contains datablock(s) I. DOI: 10.1107/S2056989025004189/nx2025Isup2.hkl

Supporting information file. DOI: 10.1107/S2056989025004189/nx2025Isup3.cml

CCDC reference: 2449698

Additional supporting information:  crystallographic information; 3D view; checkCIF report

## Figures and Tables

**Table 1:** Conformation-defining torsion angles (°) in **I**

Atoms	Torsion angle	Dihedral angle
C5—C4—C7—O1	−28.68 (16)	
C4—C7—C8—C9	−172.66 (10)	
O1—C7—C8—C9	4.91 (18)	
C7—C8—C9—S1	175.43 (9)	
C7—C8—C9—S2	−2.57 (16)	
C8—C9—S1—C10	0.01 (13)	
C8—C9—S2—C11	177.09 (10)	
		
Planar groups		
*4-F—Ph*/*b-MSP*		32.23 (4)

*4-F—Ph* is 4-fluoro­phenyl, atoms C1–C6, F1 and *b-MSP* is bis­(methyl­sulfan­yl)propenone, atoms C7–C11, O1, S1, S2.

**Table 2:** Close contacts (Å, °) in crystalline **I**

*D*—H⋯*A*	*D*—H	H⋯*A*	*D*⋯*A*	*D*—H⋯*A*
C2—H2⋯O1^i^	0.95	2.53	3.4530 (15)	163
C10—H10*C*⋯O1^ii^	0.98	2.50	3.4724 (16)	175
C11—H11*A*⋯S1^iii^	0.98	3.01	3.6032 (14)	120
C11—H11*C*⋯S2^iv^	0.98	3.01	3.6292 (13)	122

Symmetry codes: (i) *x* − 1, *y*, *z*; (ii) *x*, *y* − 1, *z*; (iii) −*x* + 2, −*y*, −*z*; (iv) −*x* + 2, −*y* + 1, −*z*.

**Table 3:** Experimental details

Crystal data	
Chemical formula	C_11_H_11_FOS_2_
*M*_r_	242.32
Crystal system, space group	Triclinic, *P* 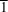
Temperature (K)	100
*a*, *b*, *c* (Å)	7.6956 (3), 8.6895 (4), 8.9468 (4)
α, β, γ (°)	74.633 (2), 83.237 (2), 73.008 (2)
*V* (Å^3^)	551.18 (4)
*Z*	2
Radiation type	Mo *K*α
μ (mm^−1^)	0.47
Crystal size (mm)	0.24 × 0.22 × 0.16

Data collection	
Diffractometer	Bruker D8 Venture dual source
Absorption correction	Multi-scan (*SADABS*; Krause *et al.*, 2015 ▸)
*T*_min_, *T*_max_	0.919, 0.971
No. of measured, independent and observed [*I* > 2σ(*I*)] reflections	15392, 2490, 2343
*R*_int_	0.030
(sin θ/λ)_max_ (Å^−1^)	0.651

Refinement	
*R*[*F*^2^ > 2σ(*F*^2^)], *wR*(*F*^2^), *S*	0.025, 0.065, 1.11
No. of reflections	2490
No. of parameters	138
H-atom treatment	H-atom parameters constrained
Δρ_max_, Δρ_min_ (e Å^−3^)	0.36, −0.20

Computer programs: *APEX5* (Bruker, 2023 ▸), *SHELXT* (Sheldrick, 2015*a* ▸), *SHELXL2019/3* (Sheldrick, 2015*b* ▸), *XP* in *SHELXTL* (Sheldrick, 2008 ▸), *SHELX* (Sheldrick, 2008 ▸) and *publCIF* (Westrip, 2010 ▸).

## supplementary crystallographic information

### 1-(4-Fluorophenyl)-3,3-bis(methylsulfanyl)prop-2-en-1-one Crystal data

**Table:**

C_11_H_11_FOS_2_	*Z* = 2
*M**_r_* = 242.32	*F*(000) = 252
Triclinic, *P*1	*D*_x_ = 1.460 Mg m^−^^3^
*a* = 7.6956 (3) Å	Mo *K*α radiation, λ = 0.71073 Å
*b* = 8.6895 (4) Å	Cell parameters from 9984 reflections
*c* = 8.9468 (4) Å	θ = 2.5–27.5°
α = 74.633 (2)°	µ = 0.47 mm^−^^1^
β = 83.237 (2)°	*T* = 100 K
γ = 73.008 (2)°	Irregular block, pale yellow
*V* = 551.18 (4) Å^3^	0.24 × 0.22 × 0.16 mm

### 1-(4-Fluorophenyl)-3,3-bis(methylsulfanyl)prop-2-en-1-one Data collection

**Table:**

Bruker D8 Venture dual source diffractometer	2490 independent reflections
Radiation source: microsource	2343 reflections with *I* > 2σ(*I*)
Detector resolution: 7.41 pixels mm^-1^	*R*_int_ = 0.030
φ and ω scans	θ_max_ = 27.6°, θ_min_ = 2.4°
Absorption correction: multi-scan (*SADABS*; Krause *et al*., 2015)	*h* = −9→9
*T*_min_ = 0.919, *T*_max_ = 0.971	*k* = −11→11
15392 measured reflections	*l* = −11→11

### 1-(4-Fluorophenyl)-3,3-bis(methylsulfanyl)prop-2-en-1-one Refinement

**Table:**

Refinement on *F*^2^	Primary atom site location: structure-invariant direct methods
Least-squares matrix: full	Secondary atom site location: difference Fourier map
*R*[*F*^2^ > 2σ(*F*^2^)] = 0.025	Hydrogen site location: difference Fourier map
*wR*(*F*^2^) = 0.065	H-atom parameters constrained
*S* = 1.11	*w* = 1/[σ^2^(*F*_o_^2^) + (0.0239*P*)^2^ + 0.2301*P*] where *P* = (*F*_o_^2^ + 2*F*_c_^2^)/3
2490 reflections	(Δ/σ)_max_ = 0.001
138 parameters	Δρ_max_ = 0.36 e Å^−^^3^
0 restraints	Δρ_min_ = −0.20 e Å^−^^3^

### 1-(4-Fluorophenyl)-3,3-bis(methylsulfanyl)prop-2-en-1-one Special details

**Table:**

Experimental. The crystal was mounted using polyisobutene oil on the tip of a fine glass fibre, which was fastened in a copper mounting pin with electrical solder. It was placed directly into the cold gas stream of a liquid-nitrogen based cryostat (Hope, 1994; Parkin & Hope, 1998). Diffraction data were collected with the crystal at 100K.
Geometry. All esds (except the esd in the dihedral angle between two l.s. planes) are estimated using the full covariance matrix. The cell esds are taken into account individually in the estimation of esds in distances, angles and torsion angles; correlations between esds in cell parameters are only used when they are defined by crystal symmetry. An approximate (isotropic) treatment of cell esds is used for estimating esds involving l.s. planes.
Refinement. Refinement progress was checked using *Platon* (Spek, 2020) and by an *R*-tensor (Parkin, 2000). The final model was further checked with the IUCr utility *checkCIF*.

### 1-(4-Fluorophenyl)-3,3-bis(methylsulfanyl)prop-2-en-1-one Fractional atomic coordinates and isotropic or equivalent isotropic displacement parameters (Å^2^)

**Table:**

	*x*	*y*	*z*	*U*_iso_*/*U*_eq_	
S1	0.73029 (4)	0.09565 (3)	0.14124 (4)	0.02169 (9)	
S2	0.86839 (4)	0.38315 (3)	0.11708 (3)	0.01940 (9)	
F1	−0.1801 (1)	0.82639 (9)	0.51997 (9)	0.02883 (18)	
O1	0.60092 (12)	0.62516 (11)	0.20784 (11)	0.02322 (19)	
C1	−0.01976 (16)	0.75951 (14)	0.44890 (14)	0.0204 (2)	
C2	−0.01958 (16)	0.65680 (14)	0.35558 (14)	0.0204 (2)	
H2	−0.128882	0.636203	0.337248	0.024*	
C3	0.14619 (16)	0.58411 (14)	0.28895 (13)	0.0179 (2)	
H3	0.151092	0.509778	0.226525	0.022*	
C4	0.30527 (15)	0.61861 (13)	0.31233 (13)	0.0164 (2)	
C5	0.29726 (16)	0.72591 (14)	0.40625 (13)	0.0189 (2)	
H5	0.404818	0.751305	0.421626	0.023*	
C6	0.13410 (17)	0.79576 (14)	0.47728 (13)	0.0209 (2)	
H6	0.128543	0.866486	0.543524	0.025*	
C7	0.48552 (15)	0.54676 (14)	0.23854 (13)	0.0170 (2)	
C8	0.51887 (15)	0.38341 (14)	0.21175 (13)	0.0175 (2)	
H8	0.421370	0.333400	0.230166	0.021*	
C9	0.68359 (15)	0.29796 (14)	0.16136 (13)	0.0162 (2)	
C10	0.52183 (19)	0.04166 (18)	0.1997 (2)	0.0401 (4)	
H10A	0.427160	0.114877	0.128935	0.060*	
H10B	0.485417	0.054133	0.305480	0.060*	
H10C	0.538334	−0.073697	0.196746	0.060*	
C11	1.05048 (17)	0.21702 (16)	0.06748 (17)	0.0281 (3)	
H11A	1.011950	0.178453	−0.013365	0.042*	
H11B	1.080620	0.125082	0.159713	0.042*	
H11C	1.157774	0.257288	0.029136	0.042*	

### 1-(4-Fluorophenyl)-3,3-bis(methylsulfanyl)prop-2-en-1-one Atomic displacement parameters (Å^2^)

**Table:**

	*U*^11^	*U*^22^	*U*^33^	*U*^12^	*U*^13^	*U*^23^
S1	0.01867 (15)	0.01518 (15)	0.03124 (17)	−0.00221 (11)	0.00121 (11)	−0.00935 (11)
S2	0.01558 (14)	0.01870 (15)	0.02352 (16)	−0.00553 (11)	0.00205 (10)	−0.00475 (11)
F1	0.0243 (4)	0.0261 (4)	0.0333 (4)	−0.0021 (3)	0.0106 (3)	−0.0129 (3)
O1	0.0202 (4)	0.0196 (4)	0.0332 (5)	−0.0087 (3)	0.0035 (3)	−0.0104 (4)
C1	0.0199 (5)	0.0162 (5)	0.0199 (5)	−0.0001 (4)	0.0050 (4)	−0.0036 (4)
C2	0.0182 (5)	0.0189 (5)	0.0235 (6)	−0.0045 (4)	−0.0002 (4)	−0.0051 (5)
C3	0.0200 (5)	0.0165 (5)	0.0182 (5)	−0.0048 (4)	0.0001 (4)	−0.0064 (4)
C4	0.0188 (5)	0.0133 (5)	0.0164 (5)	−0.0037 (4)	−0.0001 (4)	−0.0033 (4)
C5	0.0217 (6)	0.0154 (5)	0.0206 (5)	−0.0053 (4)	−0.0018 (4)	−0.0054 (4)
C6	0.0288 (6)	0.0156 (5)	0.0181 (5)	−0.0036 (5)	−0.0005 (5)	−0.0067 (4)
C7	0.0175 (5)	0.0158 (5)	0.0180 (5)	−0.0039 (4)	−0.0013 (4)	−0.0051 (4)
C8	0.0168 (5)	0.0158 (5)	0.0213 (5)	−0.0058 (4)	0.0007 (4)	−0.0062 (4)
C9	0.0170 (5)	0.0153 (5)	0.0165 (5)	−0.0043 (4)	−0.0016 (4)	−0.0039 (4)
C10	0.0246 (7)	0.0231 (7)	0.0798 (12)	−0.0105 (5)	0.0089 (7)	−0.0250 (7)
C11	0.0165 (6)	0.0223 (6)	0.0380 (7)	−0.0008 (5)	0.0055 (5)	−0.0024 (5)

### 1-(4-Fluorophenyl)-3,3-bis(methylsulfanyl)prop-2-en-1-one Geometric parameters (Å, º)

**Table:**

S1—C9	1.7421 (11)	C4—C7	1.4988 (15)
S1—C10	1.7831 (14)	C5—C6	1.3856 (16)
S2—C9	1.7478 (11)	C5—H5	0.9500
S2—C11	1.8043 (13)	C6—H6	0.9500
F1—C1	1.3626 (13)	C7—C8	1.4461 (15)
O1—C7	1.2349 (14)	C8—C9	1.3617 (16)
C1—C2	1.3740 (17)	C8—H8	0.9500
C1—C6	1.3772 (18)	C10—H10A	0.9800
C2—C3	1.3896 (16)	C10—H10B	0.9800
C2—H2	0.9500	C10—H10C	0.9800
C3—C4	1.3930 (16)	C11—H11A	0.9800
C3—H3	0.9500	C11—H11B	0.9800
C4—C5	1.3959 (15)	C11—H11C	0.9800
			
C9—S1—C10	104.24 (6)	O1—C7—C4	119.6 (1)
C9—S2—C11	103.35 (6)	C8—C7—C4	117.69 (10)
F1—C1—C2	118.27 (11)	C9—C8—C7	123.03 (10)
F1—C1—C6	118.14 (11)	C9—C8—H8	118.5
C2—C1—C6	123.57 (11)	C7—C8—H8	118.5
C1—C2—C3	117.63 (11)	C8—C9—S1	123.59 (9)
C1—C2—H2	121.2	C8—C9—S2	121.74 (9)
C3—C2—H2	121.2	S1—C9—S2	114.64 (6)
C2—C3—C4	120.99 (11)	S1—C10—H10A	109.5
C2—C3—H3	119.5	S1—C10—H10B	109.5
C4—C3—H3	119.5	H10A—C10—H10B	109.5
C3—C4—C5	119.11 (10)	S1—C10—H10C	109.5
C3—C4—C7	122.54 (10)	H10A—C10—H10C	109.5
C5—C4—C7	118.34 (10)	H10B—C10—H10C	109.5
C6—C5—C4	120.71 (11)	S2—C11—H11A	109.5
C6—C5—H5	119.6	S2—C11—H11B	109.5
C4—C5—H5	119.6	H11A—C11—H11B	109.5
C1—C6—C5	117.96 (11)	S2—C11—H11C	109.5
C1—C6—H6	121.0	H11A—C11—H11C	109.5
C5—C6—H6	121.0	H11B—C11—H11C	109.5
O1—C7—C8	122.67 (10)		
			
F1—C1—C2—C3	−177.42 (10)	C5—C4—C7—O1	−28.68 (16)
C6—C1—C2—C3	1.11 (18)	C3—C4—C7—C8	−31.93 (16)
C1—C2—C3—C4	−1.97 (17)	C5—C4—C7—C8	148.97 (11)
C2—C3—C4—C5	1.02 (17)	O1—C7—C8—C9	4.91 (18)
C2—C3—C4—C7	−178.08 (10)	C4—C7—C8—C9	−172.66 (10)
C3—C4—C5—C6	0.87 (17)	C7—C8—C9—S1	175.43 (9)
C7—C4—C5—C6	−179.99 (10)	C7—C8—C9—S2	−2.57 (16)
F1—C1—C6—C5	179.24 (10)	C10—S1—C9—C8	0.01 (13)
C2—C1—C6—C5	0.71 (18)	C10—S1—C9—S2	178.14 (8)
C4—C5—C6—C1	−1.71 (17)	C11—S2—C9—C8	177.09 (10)
C3—C4—C7—O1	150.42 (11)	C11—S2—C9—S1	−1.08 (8)

### 1-(4-Fluorophenyl)-3,3-bis(methylsulfanyl)prop-2-en-1-one Hydrogen-bond geometry (Å, º)

**Table:**

*D*—H···*A*	*D*—H	H···*A*	*D*···*A*	*D*—H···*A*
C2—H2···O1^i^	0.95	2.53	3.4530 (15)	163
C10—H10*C*···O1^ii^	0.98	2.50	3.4724 (16)	175
C11—H11*A*···S1^iii^	0.98	3.01	3.6032 (14)	120
C11—H11*C*···S2^iv^	0.98	3.01	3.6292 (13)	123

Symmetry codes: (i) *x*−1, *y*, *z*; (ii) *x*, *y*−1, *z*; (iii) −*x*+2, −*y*, −*z*; (iv) −*x*+2, −*y*+1, −*z*.
